# The teratogenic effect of Triclosan on embryogenesis is attenuated by Tween 20 in *Caenorhabditis elegans*

**DOI:** 10.17912/micropub.biology.000282

**Published:** 2020-07-23

**Authors:** Youngyong Park, Matthew A. Gaddy, Mohammad A. Alfhili, Myon Hee Lee

**Affiliations:** 1 Department of Internal Medicine, Division of Hematology/Oncology, Brody School of Medicine at East Carolina University, Greenville, NC 27834, United States; 2 Department of Clinical Laboratory Sciences, College of Applied Medical Sciences, King Saud University, Riyadh, Saudi Arabia

**Figure 1. TCS-induced embryo shrinkage and mortality is abolished by Tween 20:**
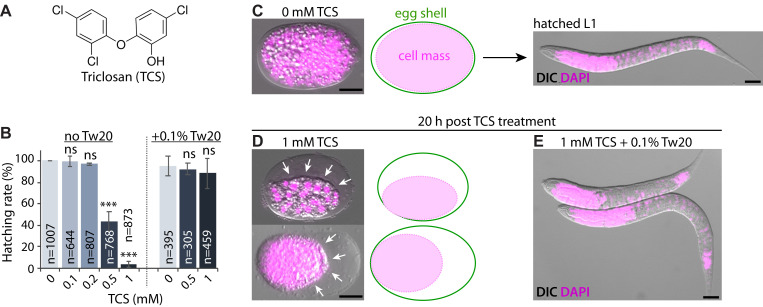
(A) Molecular structure of TCS. (B) Hatching rate of wild-type (N2) embryos at varying TCS concentrations in the presence and absence of Tween 20 (Tw20). ***, p<0.001; ns, not significant. (C-E) DAPI staining. (C) N2 embryos (~250-cell stage; a midstage embryo just before morphogenesis) and subsequent hatching visualized with DAPI (pink). (D) N2 embryos exposed to TCS show shrinkage of inner cell mass and mortality. Arrows indicate shrinkage. (E) Tw20 abrogates TCS-induced impaired hatching. Scale bar, 10 µm.

## Description

Triclosan (TCS) is a chlorinated, phenolic antimicrobial (Fig. 1A) widely used in personal care products, such as antiseptics and disinfectants, and as an additive in cosmetics, household cleaners, plastics, paints, and textiles, among others (Alfhili and Lee 2019). Despite restricted commercial use in the USA, TCS is still detected at very high rate in human samples (Weatherly and Gosse 2017), possibly because of increased use in building materials, or older polypropylene copolymers (PPCs). TCS has raised concerns regarding its health risks and environmental impact (Alfhili *et al.*. 2019; Weatherly and Gosse 2017; Yueh and Tukey 2016). In terms of risk assessment, *C. elegans* has been a successful animal model for toxicological profiling as it allows for monitoring of vital physiological endpoints such as body length, locomotion, development, brood size, and survival (Meyer and Williams 2014). We have previously shown that TCS disrupts SKN-1/Nrf2-mediated oxidative stress response in *C. elegans* larvae (Yoon *et al.*. 2017). Here, we examine the hatching rate of *C. elegans* wild-type (N2) embryos in response to acute TCS exposure (see *Methods*). As shown in Fig. 1B-1D, TCS caused pronounced shrinkage of the inner cell mass of embryos in a dose-dependent fashion. As the inner cell mass shrinks, we speculate that TCS may disturb the osmotic regulation of the developing embryo. Of note, we have recently reported that non-ionic surfactants antagonize the toxicity of phenolic endocrine-disrupting chemicals in *C. elegans* larvae (Alfhili *et al.*. 2018). Likewise, the non-ionic surfactant polysorbate 20 (also known as Tween 20; Tw20), significantly ameliorated TCS-induced mortality and restored hatching to physiological rates (Fig. 1B and 1E). Since hydrophobic substances may be emulsified in micelles formed by non-ionic surfactants, we suggest that Tw20 may inhibit TCS-induced embryonic mortality by micellar solubilization.

## Methods

*C. elegans* wild-type Bristol isolate (N2) worms were obtained from the Caenorhabditis Genetics Center (CGC). Toxicity assay was conducted using TCS and Tw20. Embryos were obtained from adult wild-type (N2) worms using 5 mL alkaline-bleaching solution as per (Yoon *et al.*. 2016). The embryos were treated with 0, 0.1, 0.2, 0.5, and 1 mM TCS in the absence or presence of 0.1% Tw20 for 1 hour at 20°C. TCS-treated embryos were washed three times with M9 buffer solution to remove remaining TCS and then incubated in M9 buffer solution with or without Tw20 at 20°C for 20 hours. The hatching rate was determined by scoring hatched, L1-staged larvae and dead embryos under a dissecting microscope. The morphology of control and TCS-treated worms was examined by DAPI staining. TCS-treated worms were fixed in 3% Paraformaldehyde for 20 minutes at 20°C and post-fixed in cold 100% methanol for 5 minutes at 20°C. The fixed worms were washed three times with 1x PBST solution and stained with 100 ng/mL DAPI for 10 minutes at 20°C. The morphology of DAPI-stained worms was observed under a fluorescence microscopy.

## Reagents

Triclosan (5-Chloro-2-(2,4-dichlorophenoxy) phenol; TCS) and Tw20 (Polysorbate 20) were purchased from Sigma Aldrich (MO, USA).

1. Alkaline-bleaching solution: 75 mL H_2_O, 20 mL commercial bleach, 5 mL 10 N NaOH.

2. TCS stock and working solutions: TCS was dissolved in ethanol as a solvent (100 mM stock). TCS stock solution was diluted to working solutions of 0.1, 0.2, 0.5, and 1 mM in M9 buffer solution with or without 0.1% Tw20.

3. M9 buffer: 3 g KH_2_PO_4_, 6 g Na_2_HPO_4_, 5 g NaCl, and 0.25 g MgSO_4_ per liter.

4. 3% paraformaldehyde fixation solution: 16% EM grade paraformaldehyde (EM Sciences) is diluted to a final concentration of 3% in 0.1 M K_2_HPO_4_ (pH7.2).

5. 1x PBST (10 mL): 1 mL 10x PBS, 0.01 mL 100% Tw20, 8.99 mL H_2_O.
